# Clinical Utility and Future Applications of PET/CT and PET/CMR in Cardiology

**DOI:** 10.3390/diagnostics6030032

**Published:** 2016-09-02

**Authors:** Jonathan A. Pan, Michael Salerno

**Affiliations:** 1 Departments of Medicine Cardiology Division, University of Virginia Health System, Charlottesville, VA 22903, USA; jap6fw@virginia.edu; 2 Department of Radiology and Medical Imaging, University of Virginia, Charlottesville, VA 22903, USA; 3 Department of Biomedical Engineering, University of Virginia, Charlottesville, VA 22903, USA

**Keywords:** positron emission tomography, cardiovascular magnetic resonance, computed tomography, PET/CT, PET/CMR, myocardial, molecular imaging

## Abstract

Over the past several years, there have been major advances in cardiovascular positron emission tomography (PET) in combination with either computed tomography (CT) or, more recently, cardiovascular magnetic resonance (CMR). These multi-modality approaches have significant potential to leverage the strengths of each modality to improve the characterization of a variety of cardiovascular diseases and to predict clinical outcomes. This review will discuss current developments and potential future uses of PET/CT and PET/CMR for cardiovascular applications, which promise to add significant incremental benefits to the data provided by each modality alone.

## 1. Introduction

Recent developments in non-invasive cardiovascular imaging, particularly advances in magnetic resonance imaging (MRI), computed tomography (CT) and positron emission tomography (PET), have provided new methods to assess myocardial structure and function. In the last few years, there has been significant progress in the development of hybrid imaging techniques, which can provide complementary imaging data from multiple modalities at the same time in a single examination [1]. Combined PET/CT allows simultaneous imaging of anatomy from CT and physiology from PET. The integration of these two systems is a natural fit since CT measures X-ray attenuation, which characterizes tissue density, providing the data necessary for attenuation correction of PET imaging. Initially intended for use in clinical oncology, PET/CT scanners have exploded in availability worldwide, opening the door for the development of cardiovascular applications. Since then, PET/CT has established itself as a powerful tool for the evaluation of patients with known or suspected coronary artery disease (CAD). By combining PET myocardial perfusion imaging (MPI) and coronary computed tomography angiography (CCTA), the extent of stenosis and the severity of obstructed blood flow can be directly compared [2]. In addition, CT coronary artery calcium (CAC) scoring and/or CCTA can be used in combination with PET radiotracer uptake, such as ^18^F-fluorodeoxyglucose or sodium ^18^F-fluoride, to assess inflammation [3], providing improved characterization of atherosclerotic plaque and risk stratification in patients. 

In the last few years, studies have emerged exploring the potential of hybrid PET/MRI systems [4,5,6]. The development of this hybrid modality lagged behind PET/CT because of major technical challenges that needed to be addressed, including interference and crosstalk between systems and attenuation correction without CT [5]. Originally developed for neuroimaging [7,8], PET/MRI is an exciting new modality for cardiovascular applications. Cardiovascular magnetic resonance imaging (CMR) uses MRI to assess cardiac function and distinguish myocardial tissue types [9]. CMR has excellent soft tissue characterization that would complement PET molecular imaging, particularly in understanding infarct viability, ventricular remodeling, inflammatory processes and infiltrative diseases. In addition, PET/CMR can be used to cross-validate new imaging techniques and study novel cardioprotective therapies. The objective of this article is to review current research and to discuss potential future applications. 

## 2. Technical Aspects of Hybrid Integration

### 2.1. PET/CT Technology

Hybrid PET/CT scanners have become widely available due to their synergism, integrating structural and physiological information. In particular, CT imaging provides the spatial data needed for attenuation correction during PET image reconstruction. During the β+ decay of the radionuclide, a positron is emitted and travels a short distance. After the positron encounters an electron, the pair undergo annihilation and produce two 511-keV photons moving in opposite directions, which can be detected by coincidence counting by the detectors. Attenuation occurs in PET imaging when the 511-keV photons emanating from deep structures in the body are absorbed or scattered before detection. Consequently, the two photons released during annihilation are unable to be detected simultaneously as a true event. Therefore, structures on the surface will show higher activity compared to more central structures. Because contrast is dependent on the degree of X-ray attenuation, CT is an ideal modality for quickly creating a map of attenuation coefficients (μ-map) [10]. 

Current PET/CT scanners have single-source CT components with slice detectors ranging from 4 to 128. Therefore, CT can also be used for CAC scoring or CCTA, which require greater than 16 and 64 slice detectors, respectively [11,12]. These protocols must be performed separately from the attenuation correction scan due their various requirements. For example, a CCTA study necessitates adequate β-blockade and intravenous contrast and, therefore, must be performed after the PET stress study [13,14]. 

The biggest challenge facing PET/CT is misalignment during attenuation correction. The problem stems from the difference in acquisitions times; PET is acquired during free breathing over multiple respiratory cycles, whereas CT is acquired in a breath-hold that is typically a few seconds. Manual alignment is currently the standard for clinical practice. Previous studies have shown that the rate of misregistration is significant and may lead to clinically-relevant artifacts in up to 40% of cases [15,16,17]. The development of strict guidelines for manual alignment and implementation of automated registration will be necessary to mitigate this issue.

### 2.2. PET/CMR Technology

Integrating PET and CMR imaging systems has been a difficult task due to interfering crosstalk between the two imaging platforms. MRI affects PET imaging in three ways. First, the high static magnetic fields prevent normal operation of the photomultiplier tubes in the PET detector. Second, rapidly changing gradient fields induce eddy current loops in PET circuitry, interfering with the signal and increasing the temperature through mechanical vibrations. Third, radiofrequency pulses can also disrupt PET-related electronics, such as high frequency clocks. On the other hand, PET scanner components can create inhomogeneity in the main magnetic field and be a source of unwanted radiofrequency energy that distorts the MRI signal acquired by the receiver coil [18,19,20]. Fortunately, most of these problems have been addressed with innovative solutions.

**Figure 1:**
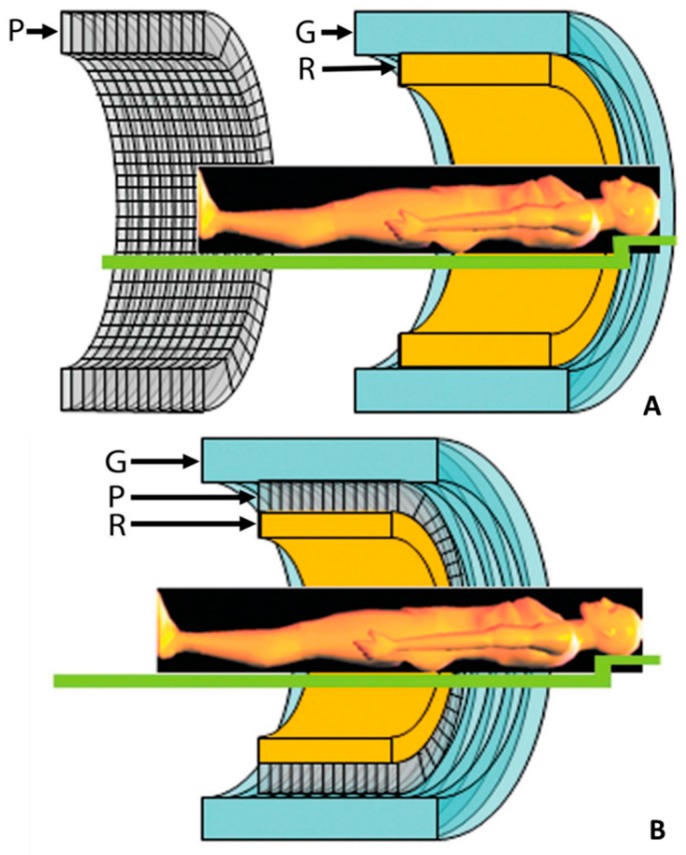
Schematic cross-sectional views of potential designs for combined PET/MR imaging systems: (**A**) sequential design with two imagers mounted from end to end and (**B**) fully-integrated design with the PET imager (P) in between the radiofrequency coil (R) and the gradient set (G) of the MR imager (**bottom**). Adapted from Torigian et al., 2013, by permission of *Radiology* [23].

Aside from software approaches that fuse images after acquisition, PET/MRI systems are designed so that patients are imaged either sequentially or in parallel (Figure 1). The Ingenuity TF scanner by Philips Healthcare’ and the trimodality system by GE Healthcare both have sequential PET/MRI designs. The Philips’ design consists of an MRI scanner followed by a PET scanner connected by a continuous gantry all within the same room. The PET detectors are shielded from the magnetic fringe field and repositioned for minimal magnetic interference. In addition, all PET electronics are removed from the room to prevent RF interference [21,22]. GE’s design has a PET/CT and an MRI scanner in separate rooms from which the patient can be transported using a mobile bed, allowing for trimodal imaging. This system minimizes the interference between systems and maximizes workload, but has the greatest risk of motion. In addition, CT is used for attenuation correction [23,24]. 

The Siemens mMR whole body integrated simultaneous PET/MRI is the first clinical scanner to acquire images simultaneously without moving the patient. In this design, PET detectors are placed between the body and gradient coils of the MRI. These detectors use lutetium oxyorthosilicate crystals coupled with avalanche photodiodes, which are insensitive to magnetic field [19]. GE Healthcare recently released their own model, the SIGNA PET/MR. Instead of avalanche photodiodes, their system uses silicon photomultiplier detectors with lutetium-based scintillators, a setup that also operates well under magnetic fields [18,25].

MRI-based attenuation correction is an ongoing challenge, as the images cannot be easily converted to μ-maps like with CT. MRI signals depend on proton density and magnetization relaxation properties, which do not confer enough information to assign attenuation coefficients to tissue types. For example, bone and air-filled cavities produce similar MRI signal intensities despite having completely different attenuation coefficients. There are two main approaches to MRI-based attenuation correction. The first approach is an atlas-based method that takes multiple MRI-CT templates from a database and registers them to the patient’s MRI. These templates are then averaged to create a pseudo-CT image, which can be converted to a μ-map [26,27]. The second approach involves the segmentation of the MRI image into different classes (lungs, fat, soft tissue and background). Adipose and water tissue can be distinguished with a Dixon sequence [28,29]. The different segments are then assigned linear attenuation coefficients based on tissue classes in order to generate a μ-map. As compared to PET/CT, the MRI attenuation algorithm resulted in small differences in measured standardized uptake values (SUV), with the largest differences in bone lesions, but these small discrepancies did not affect clinical interpretation [29].

## 3. Radiotracers

PET imaging is completely dependent on radiotracers for tracking biologic pathways in the body. The type of radiotracers chosen depends on many factors, including biodistribution, tissue target, radioisotope properties and availability. The endless possibilities for novel radiotracers make PET imaging a powerful modality for understanding cardiac physiology and disease processes. This section will overview important cardiac PET radiotracers used for research and clinical practice. 

### 3.1. PET Myocardial Perfusion Tracers

Of the available PET perfusion tracers in Table 1, ^15^O-water is considered the most ideal perfusion tracer in terms of physiologic properties. Generated by a cyclotron, ^15^O-water is freely diffusible and has a high first-pass extraction of 95%. The tracer also has nearly linear uptake with no roll-off in extraction at higher coronary flows, which would result in underestimation of myocardial flow [30]. However, with a short half-life of 123 s and poor tissue accumulation, ^15^O-water is primarily used for preclinical research. In addition, ^15^O-water is currently not approved by the Food and Drug Administration for MPI [31]. 

The perfusion tracers used in clinical PET imaging include 13N-ammonia (^13^NH_3_) and 82-rubidium (^82^Rb). ^13^NH_3_ is considered the preferred tracer due to its superior pharmacokinetics and physical properties. ^13^NH_3_ enters the myocardium either passively as NH_3_ or through Na^+^/K^+^-ATPase as NH_4_^+^. The tracer is then converted and trapped intracellularly as ^13^N-glutamine [32]. This mechanism results in a very high extraction fraction of about 80% at a baseline myocardial blood flow (1 mL/min/g), which decreases non-linearly with increasing flows due to roll-off [33]. ^13^NH_3_ is capable of producing high quality images with excellent resolution due to its long half-life of 9.96 min and short mean positron path length of 0.7 mm full width at half maximum (FWHM) [34]. The main drawback with ^13^NH_3_ is the need for an onsite cyclotron and longer examination times compared to ^82^Rb due to a longer half-life. 

^82^Rb is a potassium analog made from the parent radionucleotide 82-strontium (^82^Sr) using a ^82^Sr/^82^Rb generator and does not require a cyclotron. ^82^Rb’s uptake into the myocardium is facilitated by Na^+^/K^+^-ATPase. Compared to ^13^NH_3_, ^82^Rb has a lower first pass extraction fraction of 60% at baseline myocardial blood flows and suffers from greater roll-off at high flows [33]. In addition, ^82^Rb’s longer mean positron range of 2.6 mm FWHM attributes to a lower spatial resolution and image quality [35]. Advantages include not needing onsite cyclotron and an ultra-short half-life of 76 s that allows for rapid sequential perfusion imaging [36]. However, the cost of the generator, which is only distributed by Cardiogen-82^®^, can be prohibitive, except in high-volume centers.

In the last couple of years, 18-fluorine (^18^F)-labeled perfusion tracers have garnered much interest due to their long half-life, high myocardial extraction fraction and excellent spatial resolution. With a half-life of 110 min, ^18^F-based tracers can be acquired from regional cyclotrons, which is not possible with ^13^NH_3_. A short mean positron range of 0.2 mm FWHM allows for high image resolution. ^18^F-Flurpiridaz is the most studied tracer of this class with many promising animal studies [11,37,38,39,40]. ^18^F-Flurpiridaz is an inhibitor of mitochondrial complex-1 of the electron transport chain by competing with ubiquinone for binding sites. Targeting the mitochondria, ^18^F-flurpiridaz demonstrates a favorable biodistribution profile in rat cardiac myocytes in vitro without affecting their viability [40]. Furthermore, the radiotracer has a high first pass extraction fraction of 94% in rats independent of flow [41]. Although not approved for clinical use, phase II clinical trials have already demonstrated promising results in terms of the safety and feasibility of ^18^F-flurpiridaz [42]. 

### 3.2. Myocardial Metabolism and Viability

**Figure 2:**
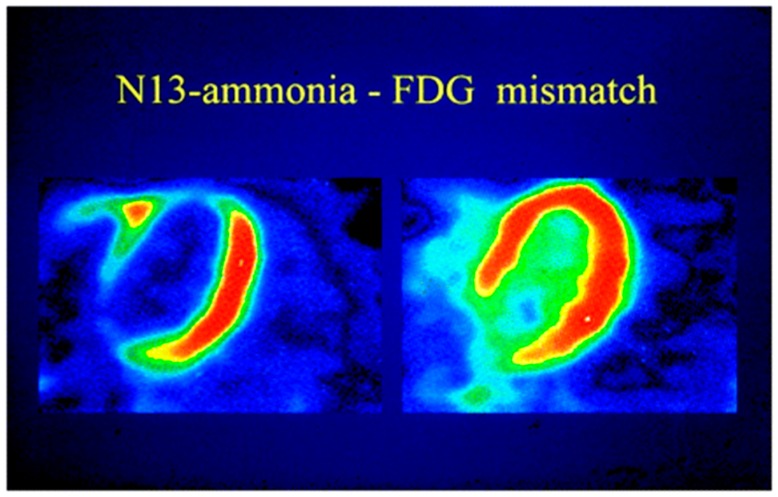
Viability imaging with PET using ^13^NH_3_ and ^18^F-FDG. Viable tissue can be identified based on the mismatch between reduced myocardial blood flow seen by poor ^13^NH_3_ uptake (**left**) and normal myocardial metabolism of ^18^F-FDG (**right**). Reproduced from Schinkel et al., 2007 by permission of *J. Nucl. Med.* [50].

^18^F-Fluorodeoxyglucose (FDG) is the most widely-used tracer for assessing metabolic activity and myocardial viability. A glucose analog coupled with ^18^F, the tracer enters myocytes via glucose transporters and is phosphorylated by hexokinase into ^18^F-FDG-6-phosphate, metabolically trapping the tracer [43]. Therefore, ^18^F-FDG has often been used to detect inflammatory, infectious and malignant foci due to their upregulation of metabolic activity [44,45,46,47,48]. To detect myocardial viability, the patient fasts for 6–12 h followed by a glucose load to induce a hyperinsulinemic state. Patients are imaged with perfusion radiotracers and ^18^F-FDG separately at rest. Both tracers will have normal uptake in healthy myocardium and be reduced in nonviable scar. Viable tissue, however, will have a mismatch in which metabolic uptake is preserved despite a perfusion defect and is more likely to benefit from revascularization (Figure 2) [49].

### 3.3. Inflammation and Plaque Characterization

The two main radiotracers used to evaluate atherosclerotic coronary plaques for inflammation and instability include ^18^F-FDG and ^18^F-fluoride. The mechanism of ^18^F-FDG was discussed in Myocardial Metabolism and Viability, but a similar idea can be applied to detect the recruitment of metabolically-active macrophages in coronary plaque [51]. Accumulation and activation of macrophage in plaque increases the likelihood of rupturing through the release of proteases that break down collagen in the fibrous cap [52]. To minimize ^18^F-FDG uptake by the myocardium, patients are given a diet low in carbohydrate and high in fat followed by 12 h of fasting before ^18^F-FDG injection [53]. Because cardiac myocytes favor free fatty acids for energy, they will suppress their glucose metabolism when flooded with their preferred energy source from adipose tissue [51,54].

Sodium ^18^F-fluoride is another radiotracer recently used by Joshi et al. [55] to identify both vulnerable and ruptured plaque based on vascular calcification. The mechanism is suggested to be related to increased osteogenic activity in the early stages of atherosclerosis and inflammation. Microcalcifications consist of hydroxyapatite deposit on the extracellular matrix and compromise the integrity of the fibrous cap. Fluoride mimics hydroxyl ions that are incorporated into the hydroxyapatite crystals on the extracellular matrix of the endothelium. They can be exchanged with one another on the crystal surface of hydroxyapatite. This process is accelerated when the surface area of the hydroxyapatite crystal reaches a critical mass, resulting in the rupture or imminent rupture of the plaque, visualized as increased ^18^F-fluoride uptake on PET [55,56,57].

### 3.4. New Tracer Options

There are a vast number of new radiotracers currently being investigated. Below are several that show promise for clinical applications in PET imaging. ^11^C-Metahdyroxephedrine (^11^C-HED) is a norepinephrine analog that targets the sympathetic nervous system of the heart, similar to 123-I-Metaiodobenzylguanidine (MIBG) for single-photon emission computed tomography (SPECT) [58,59]. Transported into sympathetic neurons by norepinephrine transporters, ^11^C-HED distributes uniformly within the myocardium and can be used to detect regional neuronal defects or denervation. Poor uptake and retention is especially noted in patients with infarctions, multivessel coronary artery disease and heart failure [42,60,61,62]. ^18^F-galacto-cyclo (RGDfK) (^18^F-galacto-RGD) is another novel radiotracer used for molecular imaging of infarct repair and healing. This radiopharmaceutical targets α_v_β_3_ integrin, a cell-membrane glycoprotein receptor that activates during angiogenesis after acute events, such as a myocardial infarction [63]. Upregulation of α_v_β_3_ and high levels of ^18^F-galacto-RGD uptake in areas of perfusion defect early after MI were associated with less left ventricular remodeling and greater angiogenesis. The ability to quantify the level of infarct repair and healing may help prognosticate patients, as well as monitor novel therapies [64,65,66].

## 4. PET-CT Studies

### 4.1. MPI and Diagnostic Accuracy of CAD

PET MPI is an excellent tool for diagnosing CAD due to its optimal combination of sensitivity and specificity. A meta-analysis from Nandalur et al. [67] looking at studies from 1977–2007 reported a sensitivity of 92% and specificity of 85% for PET MPI. These results were based on stenoses >50% in diameter seen on invasive coronary angiography. Comparing pooled diagnostic odds ratio between different MPI modalities, PET (36.47) and CMR (26.42) were found to be significantly better than SPECT (12.66) in diagnosing obstructive CAD [68]. CCTA has also been studied for its diagnostic potential for ruling out functionally-significant stenosis. In a meta-analysis by Gonzalez et al. [69], CCTA demonstrated a high sensitivity and negative predictive value (NPV) of 92% and 87%, respectively. However, CCTA only had moderate specificity and positive predictive value (PPV) of 43% and 57%, respectively. CT-perfusion and CT-fractional flow reserve had similar sensitivity and NPV and would unlikely add diagnostic value in excluding significant stenoses. Pooled data from three studies [70,71,72] have shown that combined PET/CCTA has greater diagnostic value than each stand-alone PET MPI and CCTA. By incorporating PET MPI with CCTA, hybrid imaging improved the specificity (91%) and PPV (87%) while retaining its superior sensitivity (93%) and NPV (95%) [2]. In addition, CCTA can assess the extent of atherosclerosis, which PET MPI often fails to detect in subclinical disease. Quantitative PET MPI by absolute myocardial blood flow (MBF) and myocardial flow reserve (MFR), the ratio of peak flow to resting flow, offers great potential for measuring the severity of perfusion defects and identifying balanced hypoperfusion in diffuse CAD and multivessel coronary artery stenosis. However, hyperemic MFR only shows functional flow reductions and cannot differentiate epicardial coronary artery stenosis from microvascular disease [73,74,75]. In addition, absolute MBF is limited in clinical use due to a lack of agreement on optimal cut-off values for the different tracers and its dependences on patient risk factors. A few studies suggest that CCTA can improve the diagnostic power of quantitative MPI by determining the degree of focal stenosis and differentiating it from microvascular disease [71,76,77].

### 4.2. Risk Stratification and Management

PET/CT is an excellent modality for guiding management in patients with CAD. Hybrid imaging provides complementary, rather than overlapping prognostic information. While CT can identify coronary stenoses, PET MPI can verify whether they are flow-limiting or clinically significant. The literature has shown that PET MPI can help with prognostication and risk stratification. Patients with normal MPI have good expected outcomes, while those with increasing severity of perfusion defects had proportional increases in predicted mortalities [78,79,80,81]. In 2013, Dorbala et al. [82] conducted a multicenter study with 7061 patients that showed that PET stress MPI with ^82^Rb provided significant incremental value over clinical variables. In addition, PET MPI used in combination with clinical risk markers led to significant risk restratification in 12% of patients [82]. PET quantification of MBF and MFR also adds incremental prognostic value for patients with suspected CAD [83,84,85], especially in predicting three-vessel CAD [75,86] and evaluating for microvascular dysfunction [87]. Aside from diagnosing balanced flow reductions and subclinical ischemia, quantitative perfusion may be essential for assessing disease progression and guiding management. A study [88] looking at the progression of coronary atherosclerosis using an automated semi-quantitative imaging approach demonstrated that net changes in perfusion defects in an entire coronary vascular tree predicted coronary events as opposed to changes in any one segment, including the worst flow-limiting stenosis at baseline. Therefore, quantitative MPI may be an important avenue for accurately following the progression of CAD. Finally, ECG-gating of MPI can also be used to measure left ventricular ejection fraction (LVEF) reserve. In healthy subjects, LVEF increases during peak vasodilator stress. However, in patients with CAD, LVEF decreases from baseline to peak stress in relation to increasing perfusion defects seen on stress [89]. Changes in the LVEF reserve can also be of use for risk stratification [80,81].

**Figure 3:**
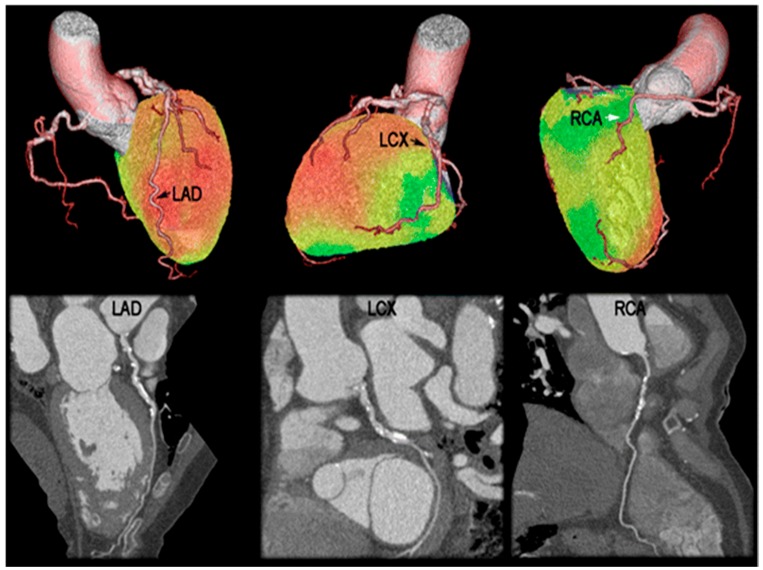
PET/CT assessing perfusion and coronary artery anatomy. Hyperemic MBF by PET (**top**) was reduced in the territories supplied by the left circumflex artery (LCX) and right coronary artery (RCA) (1.8–1.9 mL/min/g), but normal in the left anterior descending (LAD) arterial territory (2.6 mL/min/g). CCTA interpretation (**bottom**), however, suggested significant stenoses in the LAD, LCX and RCA. Adapted from Thomassen et al., 2013, by permission of *Eur. J. Nucl. Med. Mol. Imaging* [96].

CCTA complements PET MPI because it provides the anatomical pathology underlying the functional perfusion assessment. As seen in Figure 3, CCTA can be used to identify more extensive CAD, clarify equivocal findings on PET MPI and characterize plaque morphology. A large cohort study by Bittencourt et al. [90] showed that CCTA enhances risk assessment based on the extent of plaque detected, regardless of whether obstructive or non-obstructive disease is present. In addition, traditional clinical risk assessment tools, such as the Framingham Risk Score, do not accurately predict the coronary atherosclerotic plaque burden seen by CCTA [91]. Therefore, CCTA’s potential contribution to hybrid imaging is identifying the subset of patients with significant plaque burden without traditional risk factors or flow-limiting stenosis, which cause perfusion defects. CT can also be used for CAC scoring, which is another means for measuring atherosclerotic burden and vascular injury [92]. CAC scoring can enhance hybrid PET/CT MPI in detecting obstructive CAD [93]. CAC is also predictive of clinical outcomes in which increasing CAC scores correlate with stepwise increased risk of adverse events in both patients with and without ischemia on PET myocardial perfusion imaging. However, the absence of coronary calcium does not completely eliminate the possibility of flow-limiting coronary artery disease [94]. A study by Choudhary et al. [95] showed that in 81 patients, CAC combined with risk assessment and CCTA can identify 50% of those who may benefit from aggressive medical management despite a normal MPI.

**Figure 4:**
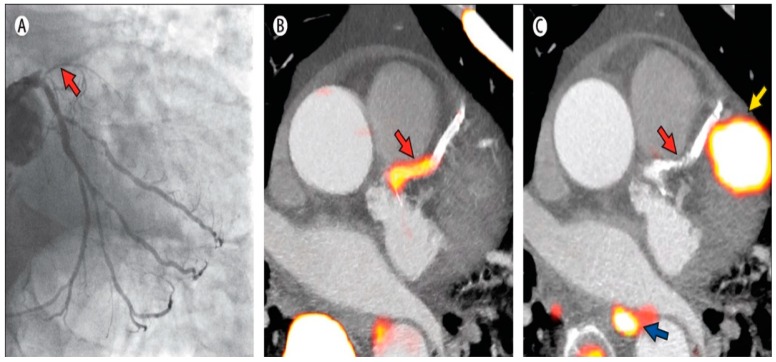
PET/CT imaging of ^18^F-fluoride and ^18^F-FDG uptake in a patient with acute myocardial infarction seen by ST-segment elevation on EKG. Invasive coronary angiography (**A**) demonstrates a proximal occlusion (*red arrow*) of the left anterior descending artery; ^18^F-Fluoride PET/CT imaging (**B**) identifies the culprit plaque (*red arrow*) based on (**B**) intense focal uptake (yellow-red); The corresponding ^18^F-FDG PET/CT image (**C**) shows no uptake at the site of the culprit. Significant uptake can be seen in myocardium next to the coronary artery (*yellow arrow*) and in the esophagus (*blue arrow*). Adapted from Joshi et al., 2014, by permission from *The Lancet* [55].

With the incorporation of radiotracers, such as sodium ^18^F-fluoride and ^18^F-FDG, hybrid PET/CT may help in staging the development of plaque and predicting plaque vulnerability. As lipid accumulates in the vessel intima, macrophages initiate an inflammatory cycle that destabilizes the plaque and forms microcalcifications in the necrotic core [57]. In later stages, however, macrocalcifications, such as those detected by CAC scoring, are believed to represent stabilized plaque in which inflammation has tempered [97,98]. PET/CT can be used to identify vulnerable plaque by evaluating for microcalcifications and inflammation using sodium ^18^F-fluoride and ^18^F-FDG [55,99,100]. Additionally, CAC scoring can rule out suspected offending plaques based on late-stage calcifications that impart stabilizing effects. Though promising, some of these studies [55,100] suggest that ^18^F-FDG has limited ability in identifying culprit plaque in coronary vessels (Figure 4). Other drawbacks of ^18^F-FDG include unintended uptake from surrounding cardiomyocytes, motion artifacts due to cardiac and respiratory activities and limited spatial resolution with current PET/CT systems [101]. 

## 5. PET-CMR Studies

### 5.1. Stress Perfusion Imaging in CAD

CMR-perfusion imaging is based on detecting differences in signal intensity, reflecting differences in myocardial perfusion, during the first-pass of a gadolinium-based contrast agent while pharmacologically vasodilated. After intravenous injection, gadolinium contrast distributes into the blood and extracellular space. While traditional protocols cover three slices (basal, midventricular and apical), novel techniques are demonstrating the increasing feasibility and accuracy of 3D coverage [102,103]. Nevertheless, dark rim artifacts and limited spatial coverage are ongoing challenges. Unlike PET, CMR’s high in-plane spatial resolution enables the detection of subendocardial defects in early stages of ischemia. Validated by invasive coronary angiography, CMR perfusion imaging has a sensitivity of 89.1% and a specificity of 84.9% on a per patient basis for CAD [104], similar to those of PET MPI (92% and 85%, respectively). Morton et al. [105] compared quantitative CMR and ^13^NH_3_ PET MPI in forty-one patients with known or suspected CAD and found that the MPRs strongly correlated (*r* = 0.75), but absolute perfusion values were only weakly correlated (*r* = 0.37). Another group [106] conducted a preliminary study with 15 patients comparing quantitative perfusion in ^82^RB PET and CMR. Their results showed a strong correlation for MPR (*r* = 0.886). Although absolute global perfusion values were higher with CMR, the differences in absolute flows strongly correlated (*r* = 0.805) between the two modalities. These two studies suggest that a single absolute stress perfusion cutoff is currently not feasible for detection of CAD and that hybrid PET/CMR may be helpful for cross-validation of the two techniques. In addition, combined PET/CMR may help realize the synergies of hybrid perfusion imaging and find ways to take full advantage of CMR’s high spatial resolution. 

### 5.2. Viability and Infarct Assessment

The concepts of myocardial stunning and hibernation due to acute and chronic ischemia, respectively, have been well documented [107,108,109,110]. The transient loss of contractility and potential for full functional recovery makes viability a highly desirable characteristic to guide revascularization management. ^18^F-FDG PET can be used to identify cardiomyocytes with preserved metabolism despite reduced perfusion. A pooled analysis by Schinkel et al. [111] showed that ^18^F-FDG PET has a sensitivity and specificity of 92% and 63%, respectively, for predicting improvement of regional function after revascularization. Di Carli et al. [112] demonstrated that the magnitude of mismatch between flow and metabolism by PET ^18^F-FDG was linearly correlated with the magnitude of improvement in heart failure symptoms after revascularization. Another important study by Beanlands et al. [49] showed that the amount of scar based on areas of reduced ^18^F-FDG uptake was a significant independent predictor of post-revascularization left ventricular function recovery. CMR late gadolinium enhancement (LGE) imaging lends itself as an alternative method measuring scar size and transmurality with high in-plane resolution. LGE is performed by intravenously injecting gadolinium and allowing the contrast agent to distribute into the extracellular and interstitial space. Areas of scarring have significant fibrosis and increased extracellular space, resulting in the accumulation and reduced washout of gadolinium contrast. A study by Kim et al. [113] showed a significant stepwise inverse relationship between transmural infarct size by CMR as a percent of the myocardium and predicted functional recovery before revascularization. There are a few studies [114,115,116] that have performed head-to-head comparisons between ^18^F-FDG PET and LGE CMR for viability assessment that demonstrated close agreement between the two, but better scar detection with CMR due to higher spatial resolution for visualizing subendocardial infarcts. Although there is no evidence for the incremental value of combined viability imaging, investigators have demonstrated that simultaneous ^18^F-FDG PET/CMR is both feasible and comparable to PET/CT [117]. A recent study by Rischpler et al. [118] looked at 28 patients with acute myocardial infarct who underwent simultaneous PET/CMR for ^18^F-FDG uptake and LGE imaging after revascularization. They found that despite substantial agreement in detecting viable myocardium, there was an 18% discrepancy in viable segments, and PET was a better predictor of recovery in these segments.

**Figure 5:**
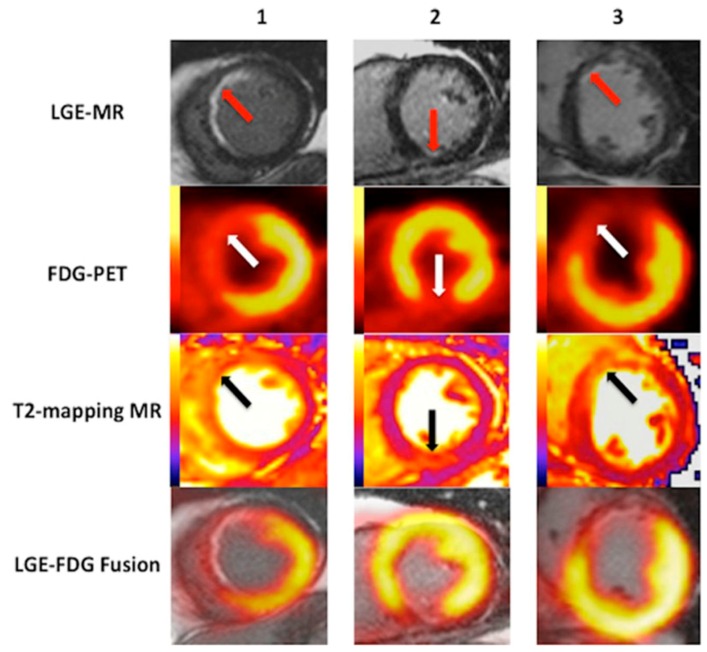
Hybrid PET/CMR imaging of three subendocardial infarctions (*red arrows*) with significant myocardial salvage. In all of the studies, the areas of reduced ^18^F-FDG uptake (*black arrows*) and of increased T2 mapping (*white arrows*) extended beyond the areas of LGE. In Study 2, the area of reduced ^18^F-FDG uptake is substantially larger than the area of LGE and more closely matched the T2 map. The follow-up scan confirms the presence of myocardial salvage, in which in area of reduced ^18^F-FDG uptake decreases in size and matches the areas of infarction. Reproduced from Bulluck et al., 2016, by permission of *Circ. Cardiovasc. Imaging* [123].

CMR can also be used to identify the extent of microvascular obstruction (MVO) and delineate the extent of myocardial edema in acute infarction. MVO adds significant independent and incremental prognostic information. In fact, some studies suggest that MVO size has the greatest prognostic value of all CMR parameters, since it reflects irreversible tissue damage and clinically-significant reperfusion injury [119,120]. Tissue edema assessed by CMR can be used as a marker of salvageable myocardium and help measure the efficacy of novel cardioprotective therapies [121]. T2 mapping techniques are commonly used to identify myocardial edema after acute coronary occlusion and ischemia [122]. A recent study by Bulluck et al. [123] using hybrid PET/CMR imaging found that reduced ^18^F-FDG uptake closely matched the area of increased T2 mapping in reperfused ST-segment elevation myocardial infarction (STEMI) patients. Interestingly, they also found that in patients with large myocardial salvage, the areas of reduced ^18^F-FDG uptake extended beyond those of LGE and closely matched the region delineated by T2 mapping, implying that reversibly injured cardiomyocyte also had impaired metabolism (Figure 5).

### 5.3. Heart Failure

**Figure 6:**
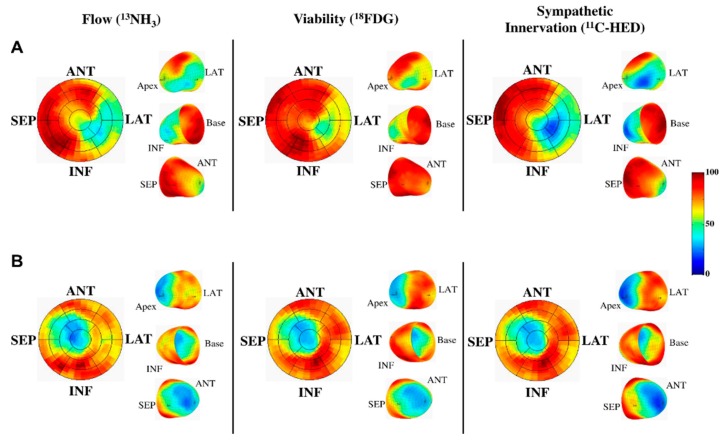
PET imaging of flow, viability and sympathetic innervation in two patients with ischemic cardiomyopathy to predict risk from sudden cardiac arrest (SCA). The subject in (**A**), who developed an SCA, demonstrated a larger volume of sympathetic denervation by ^11^C-HED compared to infarct size by ^18^F-FDG uptake. There was also reduced perfusion by ^13^NH with preserved ^18^F-FDG indicating hibernating myocardium; In contrast, (**B**) shows a subject with matched reductions in flow, infarct volume and sympathetic denervation. ANT = anterior; INF = inferior; LAT = lateral; PET = positron emission tomography; SEP = septum. Reproduced from Fallavollita et al., 2014, by permission of *J. Am. Coll. Cardiol.* [133].

Currently, echocardiography is the most commonly-used imaging test to assess ventricular function and structure in patients with suspected heart failure due to cost, accessibility and portability. However, CMR is considered a gold standard for measuring ventricular function and remodeling in heart failure due to the high spatial and temporal resolution. In addition, multiparametric CMR offers a wealth of tools to hybrid imaging in assessing the etiology and progression of heart failure [124]. CMR can accurately quantify both regional and global myocardial function and detect diffuse fibrosis. CMR tagging is a reference standard for measuring regional function, in which myocardial deformation can be visualized by a visible grid formed by nulling magnetization in two oblique planes [125]. With this method, myocardial strain can be quantified in longitudinal, circumferential and radial directions. Studies by Choi et al. [126] showed that circumferential strain added significant incremental predictive value for heart failure in asymptomatic subjects with a previous history of cardiovascular disease. Diffuse fibrosis is another potentially important marker for detecting subclinical disease and staging heart failure. Diffuse fibrosis cannot be evaluated by LGE, since there is no region of normal myocardium for comparison. Taking advantage of the fact that fibrosis is associated with the expansion of extracellular space, techniques in T1 mapping and extracellular volume (ECV) quantification have been investigated to measure diffuse collagen deposition [127]. ECV assessment [128] and T1 mapping [129] have been shown to predict adverse cardiovascular outcomes overall and in heart failure with preserved ejection fraction, respectively. Neurohormonal PET imaging can also be used to evaluate and assess sympathetic innervation in heart failure. ^11^C-HED is the most commonly-used radiolabeled catecholamine PET tracer in humans [130] The extent of reduced ^11^C-HED uptake in heart failure correlates with the patient’s New York Heart Association Functional Classification (NYHA), and ejection fraction [131] was an independent predictor for the combined end-point of death or cardiac transplantation [132] and can independently predict the risk of sudden cardiac death in ischemic cardiomyopathy (Figure 6) [133].

### 5.4. Infiltrative and Inflammatory Processes

**Figure 7:**
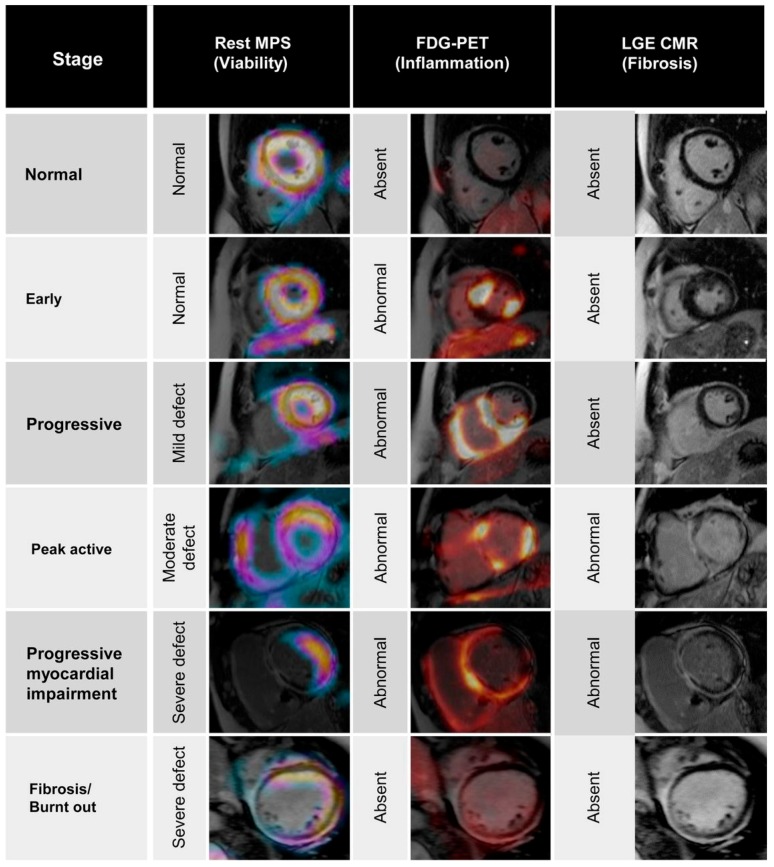
Staging of cardiac sarcoidosis using resting myocardial perfusion scintigraphy (MPS), FDG-PET and LGE-CMR. MPS shows increasing size of scarring as the disease advances. FDG-PET shows a heterogeneous pattern of inflammation in the intermediate stages, with exception to burnt-out stages in which there is no FDG uptake. LGE-CMR demonstrates the fibrotic changes, which is more prevalent in late stage sarcoidosis. Reproduced by Kouranos et al., 2015, by permission of *Br. Med. Bull.* [139].

Sarcoidosis is a systematic granulomatous disease that can lead to conduction abnormalities, arrhythmias and heart failure [134]. Currently, there is no gold standard for diagnosing cardiac sarcoidosis. CMR provides high spatial resolution and soft-tissue information for detecting the early inflammatory phase and chronic fibrotic phase. A study by Patel et al. [135] found that LGE was twice as sensitive for detecting cardiac involvement compared to the Japanese Ministry of Health and Welfare criteria and was associated with future adverse events. Recently, scar presence by CMR, even in patients with normal LVEF, has been shown to be a predictor of arrhythmia and cardiovascular death [136]. T2 imaging may also provide unique information regarding the presence of acute inflammation in cardiac sarcoid [137]. ^18^F-FDG PET can also be used to visualize inflammation in early sarcoidosis due to the upregulation of glucose transporters and glycolytic enzymes in inflammatory cells. Using myocardial ^18^F-FDG suppression protocols, PET can stage cardiac sarcoidosis based on the level of ^18^F-FDG uptake in the early phase compared to regional perfusion defects, a marker of late tissue damage [138]. PET provides more functional and biological information that can help monitor disease progression and response to therapy (Figure 7).

Cardiac amyloid is an infiltrative cardiomyopathy that may benefit from hybrid imaging. CMR with LGE can detect patterns of hyperenhancement consistent with cardiac amyloidosis in order to non-invasively diagnose cardiac amyloidosis and clarify equivocal echocardiogram findings [140]. A recent study by White et al. [141] showed that diffuse global T1 hyperenhancement by LGE accurately identifies patients with cardiac amyloidosis verified by histology and is a strong predictor of mortality. Native T1 mapping with a shortened modified look-locker inversion recovery sequence has also been used to identify cardiac amyloidosis and serves as an alternative for patients with contraindications to gadolinium contrast, particularly those with renal dysfunction [142,143]. CMR can also play an important role in characterizing amyloid tissue in order to differentiate disease subtypes, which have significantly different prognostic outcomes and treatment plans [144]. Recent studies [142,145] quantifying ECV and native T1 maps in patients with cardiac amyloidosis have demonstrated differences between the subtypes. Despite its excellent diagnostic performance for detecting cardiac amyloid, the absence of a classic LGE pattern does not definitively rule out cardiac amyloidosis, and atypical LGE patterns continue to be challenge when attempting to differentiate amyloid from other disease mimickers [146]. PET imaging may help improve the specificity of non-invasive imaging. There are currently a number of radiotracers being studied for targeting amyloid deposition, especially in the brain of Alzheimer’s patients [147]. One of these tracers, ^18^F-florbetapir, is currently being investigated in a pilot study [148] for imaging cardiac amyloidosis.

Infective endocarditis (IE) is another possible application of PET/CMR. There are limited studies evaluating the utility of CMR in IE. However, a small study by Dursun et al. [149] used multiparametric CMR to look for valvular vegetation and endothelial contrast enhancement pattern by delayed contrast-enhanced imaging. They found that CMR was able to detect valvular vegetation. Even in the absence of vegetation, CMR was useful in identifying endothelial inflammation by LGE. ^18^F-FDG PET imaging has recently been used to confirm IE diagnosis. In suspected IE patients with implanted cardiac devices [150] or prosthetic valves [151], ^18^F-FDG uptake improved the diagnostic accuracy of the modified Duke criteria. In addition, ^18^F-FDG PET is useful in visualizing peripheral embolic and metastatic infectious events [152]. However, PET imaging has poor detection of embolic events in the brain due to its high glucose use and lacks the spatial-temporal resolution to detect small oscillating valvular vegetation [152]. MRI may help overcome these obstacles and further push the diagnostic power of PET. 

Regarding plaque characterization, combined ^18^F-FDG PET/MRI has been shown to be feasible and comparable to PET/CT in the evaluation of carotid plaque [153]. However, PET/MRI coronary plaque imaging is currently limited due to cardiac and respiratory motion. Fat-MRI-based coronary motion correction techniques have been investigated to improve simultaneous ^18^F-FDG PET/MRI coronary plaque imaging [154]. In addition, the development of novel PET/MRI tracers that target activated macrophages and atherosclerotic inflammation has great potential for early detection of vulnerable plaque [154,155,156]. 

### 5.5. Molecular Imaging and Targeted Therapy

To complement volumetric and soft tissue information from MRI, PET possesses many exciting and innovative molecular radiotracers to track therapy response. ^18^F-galacto-RGD is one of many tracers investigated to image angiogenesis due to α_v_β_3_ integrin upregulation following hypoxic insult on the myocardium [157]. High ^18^F-galacto-RGD uptake in the segments with perfusion defects following a myocardial infarction was associated with the absence of significant left ventricular remodeling at 12 weeks post-myocardial infarction. These results suggest that α_v_β_3_ integrin expression is an important biomarker for monitoring myocardial repair post-infarction and may add important prognostic information [64]. ^18^F-labeled matrix metalloproteinases (MMP) inhibitors have also been developed to image both cardiac and vascular tissue remodeling, especially in advancing atherosclerotic plaque. MMP are proteolytic enzymes that are able to degrade all protein components of the extracellular matrix [158,159]. Several animal studies have already demonstrated the viability of using MMP inhibitors tagged with radiotracers to detect remodeling [160,161,162]. PET can also be used to track the effectiveness of stem cell therapy, monitoring cell survival and migration. Cells can be directly labeled by suspending them in a radiotracer solution [163]. A more advanced alternative is transducing stem cells with radiolabeled PET reporter genes via herpes simplex virus thymidine kinase [164].

## 6. Conclusions

Hybrid imaging holds immense potential for guiding the management of cardiovascular disease and progressing clinical research. PET/CT has already proven its utility in patients with CAD by fusing coronary anatomy and functional findings. PET/CMR is still in the early stages of development and validation, but offers promising clinical and research applications.

## Figures and Tables

**Table 1:** Radiotracers for PET MPI.

Radiopharmaceuticals	Half-Life (min)	Mean Positron Range (mm)	First Pass Extraction	Availability	Comments
^15^O-Water	2.07	0.9	95%	Cyclotron	Not FDA approved, linear uptake, freely diffusible
13N-Ammonia (^13^NH_3_)	9.96	0.7	80%	Cyclotron	High myocardial retention due to intracellular trapping
Rubidium-82 (^82^Rb)	1.25	2.6	60%	^82^Sr/^82^Rb Generator	Short half-life for sequential imaging, not generated from cyclotron
^18^F-Flurpiridaz	110	0.2	94%	Cyclotron	Long half-light, high extraction, no onsite cyclotron required

## Abbreviations

The following abbreviations are used in this manuscript:

PET	Positron emission tomography
CT	Computed tomography
CMR	Cardiovascular magnetic resonance
MRI	Magnetic resonance imaging
CAD	Coronary artery disease
MPI	Myocardial perfusion imaging
CCTA	Coronary computed tomography angiography
CAC	Coronary artery calcium
μ-map	Attenuation coefficient map
^13^NH_3_	^13^N-Ammonia
^82^Rb	82-Rubidium
FWHM	Full width half maximum
^82^Sr	82-Strontium
^18^F	18-Fluorine
FDG	Fluorodeoxyglucose
^11^C-HED	^11^C-Metahdyroxephedrine
^18^F-galacto-RGD	^18^F-galacto-cyclo(RGDfK)
MBF	Myocardial blood flow
MFR	Myocardial flow reserve
LVEF	Left ventricular ejection fraction
LGE	Late gadolinium enhancement
MVO	Microvascular obstruction
ECV	Extracellular volume
IE	Infective endocarditis
MMP	Matrix metalloproteinases
